# Exploring LA-ICP-MS as a quantitative imaging technique to study nanoparticle uptake in *Daphnia magna* and zebrafish (*Danio rerio*) embryos

**DOI:** 10.1007/s00216-015-8720-4

**Published:** 2015-05-06

**Authors:** Steffi Böhme, Hans-Joachim Stärk, Dana Kühnel, Thorsten Reemtsma

**Affiliations:** Department of Bioanalytical Ecotoxicology, Helmholtz Centre for Environmental Research - UFZ, Permoserstrasse 15, 04318 Leipzig, Germany; Department of Analytical Chemistry, Helmholtz Centre for Environmental Research - UFZ, Permoserstrasse 15, 04318 Leipzig, Germany

**Keywords:** Visualization, Toxicity, Accumulation, Microscopy, Tissue, Soft biological matrices

## Abstract

The extent and the mechanisms by which engineered nanoparticles (ENPs) are incorporated into biological tissues are a matter of intensive research. Therefore, laser ablation coupled to inductively coupled plasma mass spectrometry (LA-ICP-MS) is presented for the detection and visualization of engineered nanoparticles (Al_2_O_3_, Ag, and Au) in ecotoxicological test organisms (*Danio rerio* and *Daphnia magna*). While ENPs are not taken up by the zebrafish embryo but attach to its chorion, incorporation into the gut of *D. magna* is clearly visible by a 50-μm spot ablation of 40-μm-thick organism sections. During laser ablation of the soft organic matrix, the hard ENPs are mobilized without a significant change in their size, leading to decreasing sensitivity with increasing size of ENPs. To compensate for these effects, a matrix-matched calibration with ENPs of the same size embedded in agarose gels is proposed. Based on such a calibration, the mass of ENPs within one organism section was calculated and used to estimate the total mass of ENPs per organism. Compared to the amount determined after acid digestion of the test organisms, recoveries of 20–100 % (zebrafish embryo (ZFE)) and of 4–230 % (*D. magna*) were obtained with LODs in the low ppm range. It is likely that these differences are primarily due to an inhomogeneous particle distribution in the organisms and to shifts in the particle size distribution from the initial ENPs to those present in the organism. It appears that quantitative imaging of ENPs with LA-ICP-MS requires knowledge of the particle sizes in the biological tissue under study.

**Electronic supplementary material:**

The online version of this article (doi:10.1007/s00216-015-8720-4) contains supplementary material, which is available to authorized users.

## Introduction

Engineered nanoparticles (ENPs) have a huge variety of applications, while their behavior and effects in the environment are poorly known and a matter of ongoing research. Analytical methods for the determination in all environmental compartments are being developed and have to be improved as a prerequisite for studies on the fate, the uptake, and the bioaccumulation behavior of ENPs in the environment and environmental organisms, respectively.

For the determination of nanoparticle distribution in organisms or cells, visualization with optical devices is advantageous. Until now, methods such as micro-particle-induced X-ray emission (micro-PIXE) or magnetic resonance imaging (MRI) have been used [1, 2]. Other techniques often apply magnetic, fluorescent, or radio-labeled particles which may require a modification of the physical or chemical properties of the ENPs under study. For metal or metal oxide ENPs, the combination of laser ablation with element-specific detection by laser ablation coupled to inductively coupled plasma mass spectrometry (LA-ICP MS) allows their determination without the need for labeling. ICP-MS systems offer a high-sensitivity, high-precision, and wide dynamic range of up to 6 orders of magnitude [3]. LA-ICP-MS was initially established for the element detection in geological and archeological samples [4] and was later applied to determine metalloproteins and metalloenzymes after separation by gel electrophoresis [5] and to record the elemental distributions in biological tissues [6–8]. In these reports, the area studied was in the square micrometer-to-centimeter range. In order to increase the spatial resolution to a range suitable to detect single ENP and their uptake and distribution on a cellular level, the equipment as well as the software had to be improved [9–11].

When LA-ICP-MS is used not only to study the spatial distribution of elements but also to quantify their amounts, suitable calibration approaches are required. For hard geological samples, solid reference materials like glass, ceramic, and metals are available for calibration [4].

Quantification becomes more challenging from soft matrices such as biological tissues because laser ablation from such materials is less controlled [12]. A calibration approach suitable for metal ions is the use of matrix-matched standards. For example, Becker et al. [6] quantified uranium cations in rat brain sections by LA-ICP-MS using spiked rat brain tissue homogenates for calibration. The selection of a suitable matrix depends on the ablated mass and instrumental drifts during the long ablation periods (often several hours) [13]. Especially, the application of carbon as an internal standard is questionable since Frick and Günther [14] proved the separate transport of carbon in the gaseous phase and of trace elements in the particle phase. Konz et al. [13] presented an internal standard correction methodology by the application of homogenous thin gold films on the tissue surface which can enhance the optical structure definition and increase the ablation efficiency.

While strategies for the quantification of metal ions from soft tissue by LA-ICP-MS have been developed, it is not clear, yet, how quantification works in the case that hard metal ENPs are ablated from soft tissue matrices. For the reasons given above, it appears unlikely that external calibration as for solid materials or by liquid metal standards is suitable. Often studies applied LA-ICP-MS solely as a visualization technique to gain information on the ENP distribution [15–17]. In contrast, Drescher et al. [9] deposited ENPs on a nitrocellulose membrane and analyzed the spiked material for calibration of ENPs in cells by LA-ICP-MS to obtain semi-quantitative data. Koelmel et al. [18] applied AuNP-spiked cellulose pellets as standards, and Wang et al. [19] performed quantification by the ablation of droplet residues on glass slides containing Au standards. Another way is the spiking of the respective sample material with a range of NP concentrations as presented by Judy et al. [20] for tobacco plant leaves. Recently, Böhme et al. [21] spiked agarose gels with Al_2_O_3_ nanoparticles for calibration of LA-ICP-MS analysis of whole cell layers. That study showed that ENPs of different sizes may exhibit a different response. This indicates that the processes taking place during the ablation of ENPs from soft matrices and their size dependence require further investigation. If quantitative imaging by LA-ICP-MS was feasible, it would allow determining internal concentrations for specific regions or organs of small organisms and thus improve (a) to study the kinetics of uptake and internal transport and (b) to link biological effects to internal concentrations in the sensitive organ.

Therefore, this study explores the suitability of LA-ICP-MS for the quantitative imaging of ENP uptake by ecotoxicological test organisms like *Daphnia magna* and zebrafish (*Danio rerio*) embryos. For methodical investigations, agarose gels as a model for soft matrices are spiked with ENPs of different qualities (Al_2_O_3_, Ag, Au) and different particle sizes and the sensitivity of detection by LA-ICP-MS is studied. In addition, we aim to investigate the potential of this calibration approach by applying it to real organisms exposed to ENPs in lab studies and comparing the ENP concentrations determined by LA-ICP-MS with the data gathered after acid digestion of whole organisms.

## Materials and methods

### Characterization of nanoparticle suspensions

The investigated silver and gold nanoparticle suspensions were provided and characterized in detail by partners of the EU project NanoValid (see Electronic Supplementary Material (ESM) Table S1). The silver particles are characterized by a primary particle size (PPZ) of 21 ± 8 nm, a polyvinylpyrrolidone (PVP) coating, and a dissolved silver fraction of 48 %. In contrast, the gold particles are stabilized by sodium citrate to prevent agglomeration processes within the suspension and have a PPZ of 13 ± 1 nm. The three aluminum oxide nanomaterials (Al_2_O_3_-NPs) were purchased as powders from industrial partners and were characterized in detail within the study of Böhme et al. [21]. Sodium hexametaphosphate (SHMP, 0.05 % (*w*/*v*); Merck KGaA) is used as a stabilizing agent for the Al_2_O_3_ stock suspensions. In the case of Al_2_O_3_-NPs, the specific surface area was used to calculate the mean particle sizes of 14, 111, and 750 nm, respectively. Furthermore, dynamic light scattering (DLS) was applied to determine the particle size distribution and the zeta potential of the ENPs in the stock suspensions.

### Organism cultivation and sample preparation

Zebrafish embryos (*D. rerio*) were cultivated and exposed to the respective ENPs according to the OECD test guideline 236 [22]. The organisms were cultured at 26 ± 1 °C at a 14:10-h light/dark cycle. Fish were fed daily with *Artemia* sp. ad libitum. For the egg collection, spawn traps were placed into the fish tanks on the day prior to spawning. After selection of fertilized eggs, exposure experiments were started at a time point of 2 h post fertilization (hpf) in ISO water for 24 h with an ENP concentration of 100 μg element/L which is below the respective EC_50_ values (data not shown). The exposure of *D. magna* was performed according to the OECD test guideline 202 [23]. The crustaceans were cultivated under controlled conditions at 20 °C at a 16:8-h light/dark cycle. Daphnids were fed three times a week with algae, and the individuals aged less than 24 h (neonates) were exposed to the respective nanomaterial in Aachener Daphnien Medium (ADaM) for 48 h with an ENP concentration of 10 μg element/L (non-toxic). After exposure, only living, healthy organisms were collected and washed twice with a medium.

For the acid digestion of whole organisms, 10 individuals of each species were collected for one sample and a minimum of three biological replicates was performed.

For the visualization by LA-ICP-MS, whole organisms were fixated with a paraformaldehyde phosphate-buffered solution, embedded with frozen section medium (Neg-50; Richard Allen Scientific), and cut in 40-μm sections using a microtome (Microm CryoStar, HM 560; Thermo Scientific) at −20 °C. The sections were placed on glass slides, and the number of sections needed for one individual organism was noted. A minimum of three independent biological replicates was performed, and one section of each individual was analyzed.

### Determination of the total element concentrations

A quadrupole ICP-MS (ELAN DRC-e; PerkinElmer SCIEX) was applied to analyze the element concentrations of the exposed organisms, either from solution after acid digestion or from the aerosol generated by laser ablation (see below). The acid digestion of organisms exposed to Al_2_O_3_-NPs was performed with hydrochloric acid (30 %, Suprapur; Merck) and the addition of 500 mg potassium chlorate (for analysis; Merck) in separate reaction vessels for 4 h (HPA-S, Anton Paar; temperature 250 °C, pressure 100 bar) [21]. Ag and AuNPs were solubilized by digestion in an open system (DigiPrep, S-Prep) with nitric acid (65 %, Suprapur; Merck) or aqua regia (3:1 HCl:HNO_3_), respectively. An external calibration was performed by analyzing the respective element standard reference solutions (1 g/L; Merck).

### ENP visualization by LA-ICP-MS and external calibration

The exposed organism sections were ablated using a Nd:YAG laser with a wavelength of 266 nm (LSX 500; CETAC, USA) (ESM Table S2). The laser energy was adjusted to 60 % (~6 J/cm^2^) to ensure a complete ablation of the organic layer. The nebulizer was used in parallel to mix the laser aerosol with a blank solution and to have constant wet plasma conditions. Due to instrument limitations (ablation chamber with long washout times), a 50-μm spot diameter as spot size for the spot ablation was selected to avoid measurement times above 4 h for one organism section.

An external calibration was performed with individually spiked agarose gels of 40 μm in thickness [24]. Instead of dissolved metal cations, the ENPs under study were spiked to the agarose solutions after heating (90 °C) [21]. Then, 4 ml of the agarose solution was carefully pivoted and immediately deposited on the glass slide. The gel slides were allowed to dry and analyzed by LA-ICP-MS with the same laser ablation parameters as the organism sections. Both organism sections and agarose gels had a thickness of 40 μm (*z*-direction), and thus, a mass of ~100 ng per spot was ablated. The measured element intensities for the ablated gel slides were corrected by a factor of 1.141 according to the concentration gradient at the boundary of the slide [24]. The transient signals for the individual spots were collected in a data matrix. By application of the external calibration curves, the intensities were transformed to concentration values. The concentration for the scale bar of the colored contour plots was determined as a particle concentration in picograms per ablated spot. In addition, LODs of the applied method can be found in ESM Table S3.

### Aerosol particle analysis

To determine the particle size distribution of the aerosol particles generated by laser ablation, the aerosol was collected on an adhesive carbon film placed on a SEM sample carrier for 30 s. To avoid a charge-up of the sample, the surface was sputtered with gold for 20 s. The carrier was then introduced into a scanning electron microscope equipped with a field emission cathode (SEM, JEOL JSM-6300F). A voltage of 1.5 kV, a working distance of 8 mm, and a secondary electron image (SEI) mode were applied to gain the SEM images. To determine the particle size distribution, the mean was calculated over the measured particle sizes of ~50 particles.

### Data evaluation

The complex data received by LA-ICP-MS measurements were evaluated by self-written software obtained with MATLAB 13a (MathWorks, USA). Different parameters, like the number of measured elements, data offset, spot number, or the time between spots, can be adjusted. In detail, for each spot, a mean over the three largest values is obtained and the background is subtracted. Due to the knowledge on the total number of ablated spots and the number of spots in *x*- and *y*-direction a data matrix can be built. Finally, the data matrix containing all spot-related values, a first contour plot, and an overview of the measurement parameters was displayed by the software. The final contour plots were generated by OriginPro 9.1G (OriginLab Corporations, USA) due to a higher image quality. Therefore, the element intensities were transformed into concentration data using the matrix-matched calibration. The LA-ICP-MS contour plot was overlayed with a sample image taken before the analysis using an installed microscopic camera. For the comparison with the total element concentrations of whole organisms determined by digestion and subsequent ICP-MS measurement, the concentration determined from one section by LA-ICP-MS has to be extrapolated. Due to the fact that the organism section is completely ablated by the laser, all individual spot concentration values can be added and multiplied by the number of sections. For this purpose, the number of sections for one individual organism was noted before (18–20 sections).

## Results and discussion

### Visualization of ENP distribution by LA-ICP-MS

**Fig. 1:**
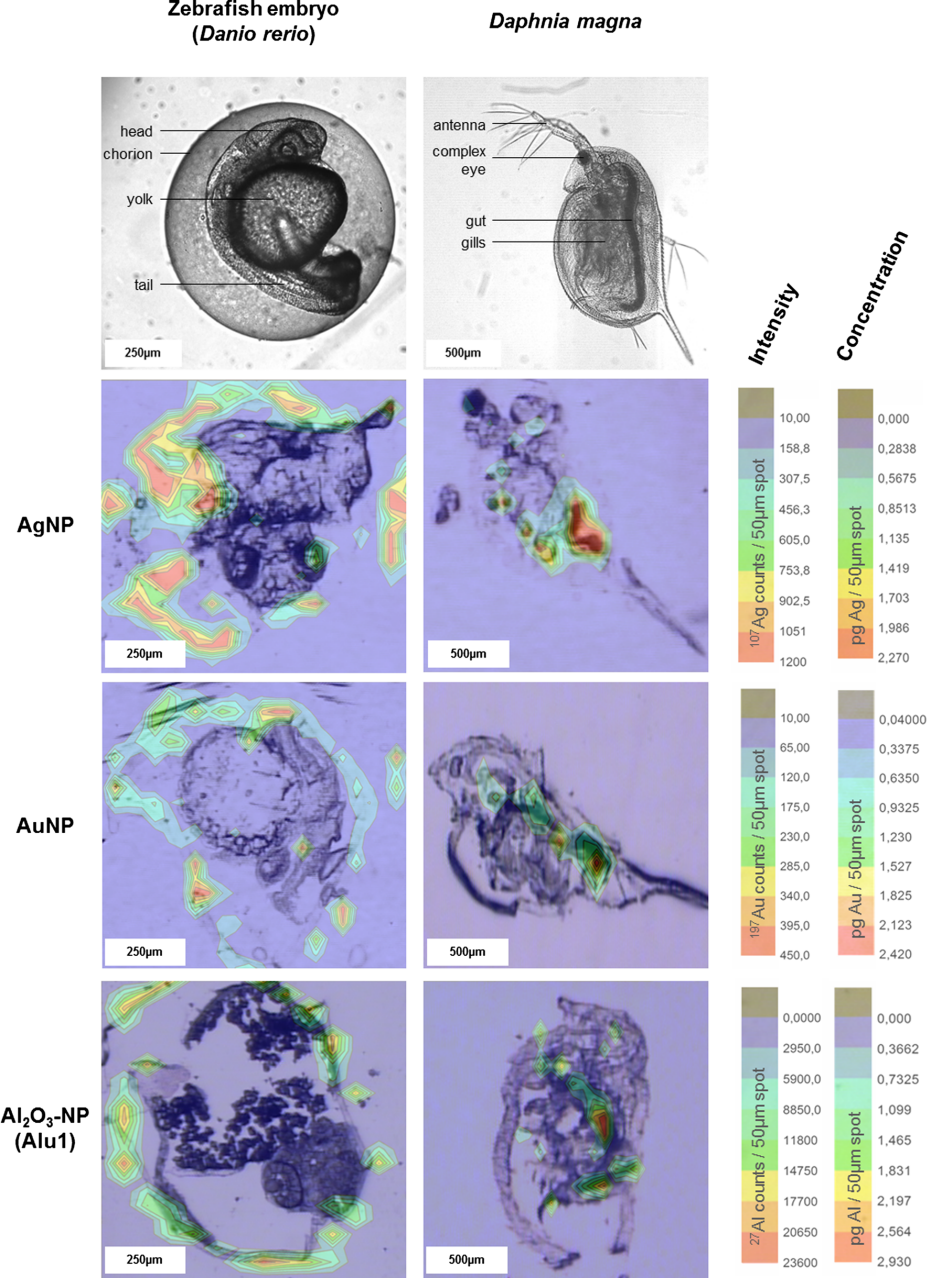
Overlay of visual images and the signal intensity for the respective element as recorded by LA-ICP-MS of ZFE (*left*) and *Daphnia magna* (*right*)

Within this study, a LA-ICP-MS method to visualize the ENP uptake and the distribution in the biological tissues of environmental organisms, like the zebrafish (*D. rerio*) embryo and a crustacean (*D. magna*), was developed. These organisms are established model systems for the toxicological testing of chemicals, and the available exposure protocols [22, 23] were adapted for nanomaterials. To visualize the uptake and to locate the regions of ENP accumulation within the organisms, a 50-μm spot size provided sufficient spatial resolution and reduced the time required for data recording compared to a smaller spot size. Moreover, larger spot sizes lead to higher sensitivity because more materials per spot are ablated (ESM Table S3). From the LA-ICP-MS data of one organism section, a 2D color plot was generated and overlayed with a visual image of the same section before ablation. These overlays clearly show an inhomogeneous ENP distribution for both organisms and all studied nanomaterials (AgNPs, AuNPs, and Al_2_O_3_-NPs), with characteristic differences (Fig. 1, ESM Fig. S1).

For zebrafish embryo (ZFE), most of the particles were accumulated at the chorion, an envelope surrounding the embryo for the first days of its development (Fig. 1). ZFE is solely nourished from the yolk within these first days of development and cannot actively take up nutrients and, even less so, particles. This particle barrier function of the chorion is already described in the literature [25, 26].

On the contrary, the ENP distribution in *D. magna* clearly indicates active uptake, since metal signals are elevated in the gut of the organisms and minor amounts are visualized to be accumulated in the gill and eye tissues (Fig. 1). This finding is in agreement to other studies where the gut uptake of fluorescent polystyrene beads [27] and CuO-NPs [28] in *D. magna* was investigated by confocal and electron microscopy.

Visualization by LA-ICP-MS, indeed, shows the regions of the test organisms where different ENPs are accumulated. In addition, LA-ICP-MS parameters were optimized to limit the analysis time with sufficient spatial resolution.

### Matrix-matched calibration approach

It appeared useful to elucidate the potential of LA-ICP-MS not only to visualize but also to quantify ENPs in biological tissues. To study the sensitivity of detection for different ENP materials as well as possible size-dependent effects, defined amounts of ENPs of Al_2_O_3_, Au, or Ag of different particle sizes were embedded in agarose gels to simulate the biological objects under study.

**Fig. 2:**
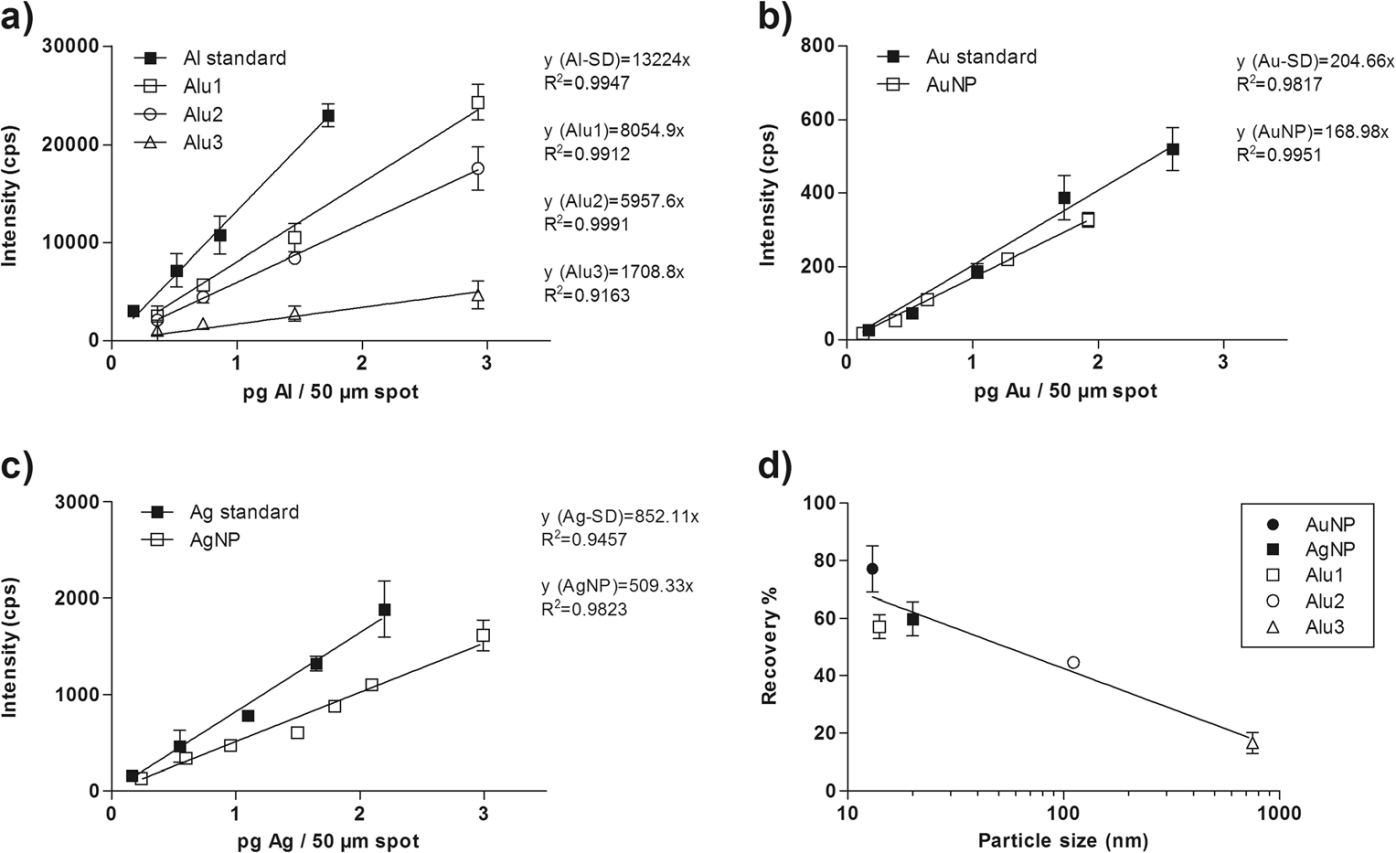
Signal intensity recorded by LA-ICP-MS for different ENPs and for metal cations (element standards) from agarose gels versus concentration of (**a**) Al_2_O_3_, (**b**) Au, and (**c**) Ag (*n* ≥ 3). Signal intensity for ENP relative to the respective metal cation (recovery) versus the ENP size is shown in (**d**)

The response obtained by spot ablation of the ENPs from agarose gels was compared with the results determined under the same instrumental conditions for agarose gels spiked with the dissolved metal cations. For all the particles under study, linear calibration curves were obtained with low standard deviation (Fig. 2). The high precision indicates that an even distribution of the ENPs in the agarose gels was achieved, which is a prerequisite for this calibration approach. For all the materials, the sensitivity of detection was lower for the ENPs compared to the respective metal cation dissolved in agarose gel. This effect was weakest for Au (Fig. 2(b)) and more pronounced for Ag (Fig. 2(c)) and Al_2_O_3_ (Fig. 2(a)). For the Al_2_O_3_-NPs, for which three particle sizes were available (mean primary particle size of 14, 110, and 750 nm), strongly decreasing signal intensities with increasing particle size were observed (Fig. 2(a)). For the largest particle size (750 nm), the signal intensity decreased to about 20 % of the Al^3+^ standard.

For the three materials under investigation, the decrease in signal intensity with increasing particle size does not seem to depend so much on the respective material (Fig. 2(d)). Up to a particle size of approx. 100 nm, the response of the ENPs remained in the range of 50–75 % and no strong size dependency is visible. Above that size, however, the sensitivity strongly decreased. This trend could not be weakened by improved tuning of the instrument, e.g., by increasing the laser energy (data not shown).

Several processes may contribute to this size-dependent sensitivity, such as (a) a size-dependent degree of evaporation of particle material out of the soft biological matrix, (b) a variable degree of formation of small aerosol particles upon ablation, (c) a size-dependent transport efficiency for the aerosol particles from the site of ablation to the plasma, and eventually, (d) a size-dependent extent of evaporation and ion formation from the aerosol particles in the plasma.

To study this in more detail, aerosol particles generated by LA-ICP-MS from Al_2_O_3_-NPs of all the three sizes from the agarose matrix were collected on an adhesive carbon film placed on a SEM sample carrier approximately half the way between the ablation chamber and the plasma. The particle size distributions of the collected aerosols measured by SEM were in a comparable range as the initial sizes of the ENPs in suspension as determined using the BET method (*x*_BET_) (ESM Table S4). Furthermore, particle agglomerates or aggregates in the aerosol were observed which even exceeded the size of the original particles (ESM Table S4). These data suggest that, in the case of the soft tissue matrix studied here, the mobilization of ENPs occurs largely by the interaction of the laser with the organic material surrounding the ENPs and that the embedded ENPs are transferred into the gas stream with only minor reduction in size. In cases where a part of the laser energy is taken up by ENPs, this may lead to their superficial melting, allowing for their agglomeration to even larger particles during the evaporation process. Such agglomeration has been described upon incomplete ablation from a hard material [29].

It has been shown before that an increasing diameter of the particles in the aerosol may lead to decreasing transport speed and transport efficiency towards the plasma by the gas stream [3, 30]. While, for the analysis of ENPs from suspension, their incorporation into aqueous droplets can increase the transport efficiency to almost 100 % [31], this approach is not feasible for LA-ICP-MS of soft biological matrices. Eventually, in the plasma, small particles may completely evaporate during their residence time, whereas larger particles (>150 nm) may evaporate only incompletely, leading to decreasing efficacy of evaporation and ionization in the plasma with increasing particle size [32, 30]. Therefore, in the case of ENP determination from soft tissue ablation, transport and ionization may be size dependent, all contributing to decreasing sensitivity for ENPs of increasing size.

More data of ENPs of different compositions and over a larger size range would be needed to assess whether the decrease of sensitivity with size compared to the dissolve metal cations is independent of the ENP material and whether a suitable equation can be derived to describe the relationship between particle size and the relative response obtained by LA-ICP-MS.

Based on the above findings, a quantification of ENPs from soft biological tissues by LA-ICP-MS requires a matrix-matched calibration with ENPs of similar size distribution as in the samples analyzed. The agarose gel matrix can be considered similar to the biological material in organic carbon and element composition as well as in water content. Moreover, the ENPs are embedded into the biopolymer rather than deposited only on its surface, similar to what one expects for the exposed organisms. This is different to the analysis of ENPs from thin cell layers, where ENPs deposited on the surface of glass [19] or filters [9] have been employed for matrix-matched calibration.

### Quantification of ENPs in ZFE and *D. magna* by matrix-matched calibration

The calibration graphs obtained from the ENPs embedded in agarose gels (Fig. 2) were used to convert the signal intensities of the LA-ICP-MS analysis of ZFE and *Daphnia* sections to the respective concentration scale (pg element/50-μm spot). From these data, the mass of ENPs per section was calculated, and from that, the total mass of ENPs per organism was estimated by multiplying it by the number of sections per organism. To test the validity of these data, the mass of ENPs in the test organism was independently determined by acid digestion and neb-ICP-MS analysis of 10 individuals (ZFE and *D. magna*). This second approach should provide information on the true ENP concentration, provided that the ENPs are fully digested (Table 1).

**Table 1:** Concentration of ENP in ZFE and *Daphnia magna* determined by acid digestion with neb-ICP-MS and calculated from the LA-ICP-MS data (*n* ≥ 3)

Nanomaterial	ZFE (*Danio rerio*)			*Daphnia magna*		
	Digestion	LA-ICP-MSx		Digestion	LA-ICP-MS	
	ng/individual		% of digestion	ng/individual		% of digestion
AgNP	5.4 ± 3.1	5.4 ± 2.9	100	0.9 ± 0.6	2.1 ± 0.5	233
AuNP	41.6 ± 18.0	8.3 ± 2.4	20	132.4 ± 33.1	5.5 ± 1.8	4
Al_2_O_3_ (Alu1)	72.5 ± 8.6	27.2 ± 3.1	38	122.9 ± 43.7	4.6 ± 2.9	4
Al_2_O_3_ (Alu2)	84.3 ± 29.1	81.3 ± 23.1	96	96.1 ± 36.2	6.9 ± 4.3	7
Al_2_O_3_ (Alu3)	92.7 ± 26.3	64.6 ± 17.4	70	92.1 ± 33.1	6.7 ± 1.8	7

The ENP concentration for the two test organisms as determined after acidic digestion by neb-ICP-MS varies from 5 to 130 ng/individual (Table 1). Such concentrations are within the range reported in the literature, where 150 ng of Al was found in *D. magna* exposed to 20 mg/L Al_2_O_3_-NPs [33] and 30 ng of Au at an exposure concentration of 0.5 mg/L AuNPs [34]. In addition, Auffan et al. [25] showed total silver concentrations of 60–140 ng/fish embryo after the exposure to 5 mg/L AgNPs.

The validity of the quantitative imaging by LA-ICP-MS with the external matrix-matched calibration using agarose gels strongly differs for the two kinds of test organisms (Table 1). For ZFE, this approach shows a median recovery of 70 % for all the materials and particle sizes, which is an acceptable result. The variability is, however, high with lowest recovery for Au (20 %) and highest for Ag (100 %). The recoveries for Al_2_O_3_ were between these two extremes. However, also here, the recovery varied from 38 to 96 %, without an obvious size dependency (Table 1). These variable recoveries can, in part, be explained by the fact that (a) not the same individual was used for either analysis and that (b) for digestion and neb-ICP-MS analysis, 10 whole organisms were measured, whereas in the case of LA-ICP-MS, the data for one organism section were extrapolated to the (one) whole organism. Thus, inhomogeneous ENP distribution will contribute to different ENP concentrations determined by neb-ICP-MS and LA-ICP-MS.

The results were much worse for *D. magna*, however, with a median recovery of only 7 % (Table 1) and a difference between 4 % for Au and 233 % for Ag. Recoveries for AgNPs of >100 % for both test organisms may be explained by the fact that Ag was partially dissolved from the ENP surface and that this part experienced much better efficiency of ablation, transport, and ionization compared to the particulate Ag. For Au and Al_2_O_3_-ENP, the recovery by LA-ICP-MS in *D. magna* was extremely poor (4–7 %). Obviously, the imaging for ENPs in *D. magna* by LA-ICP-MS did not deliver valid quantitative information with the applied matrix-matched calibration.

Some of the variability in both ZFE and *D. magna* may originate from the variability of the tissue matrix as such, which may influence the ablation process. This variability cannot be compensated by internal standardization [35], but why is the analytical approach leading to reasonable data for ZFE not suited for *Daphnia*?

In the case of ZFE, the ENPs were evenly distributed over the chorion surface (Fig. 1); this increases the probability that the amount of ENPs determined in one section is representative for and comparable to the amount of the other sections of the organism. Moreover, it is reasonable to assume that the particles adsorbed to the chorion are mostly unchanged in size. Under these conditions, the matrix-matched calibration can sufficiently compensate the size-dependent sensitivity of LA-ICP-MS (Fig. 2). As a consequence, the quantitative imaging of ENPs by LA-ICP-MS for one section is correct and the extrapolation to the whole organism provides a reasonable estimate of the amount of ENPs per ZFE organism (Table 1).

For *D. magna*, however, the images provided by LA-ICP-MS clearly show that the ENPs were ingested (Fig. 1). Since the intestine is not evenly distributed over the whole organism, the amount of ENPs in one section may significantly differ from their amount in another section. This would explain a higher variability of the concentration data gathered for the *Daphnia* organisms. Besides that, in the gastrointestinal tract of the *D. magna*, ENPs may change their shape and size by agglomeration due to the influence of enzymes or proteins within the digestion fluid. Indeed, an enhanced agglomeration of TiO_2_-NPs [36] and an active in vivo biomodification of lipid-coated carbon nanotubes through digestion by *D. magna* were already reported [37]. If these processes lead to a significant particle agglomeration and thus to increasing particle sizes compared to the original ENPs, the calibration with the initial ENP size would clearly underestimate the amount of ENPs in the tissue (Fig. 2). This would explain the poor recovery of ENPs from *D. magna* by LA-ICP-MS (Table 1).

Due to the abovementioned difficulties of ENP quantification within *D. magna*, it appears that quantitative imaging by LA-ICP-MS requires knowledge of the particle size distribution within the organism under study. This knowledge may be gathered by electron microscopy or using other analytical methods, requiring separate sample preparation and analysis. In case that the final particle size distribution in the organism significantly differs from the initial distribution of the ENP size, a matrix-matched calibration with this final particle sizes has to be performed for valid quantitative imaging.

## Conclusion

As shown here for Ag, Au, and Al_2_O_3_ nanoparticles, imaging by LA-ICP-MS allows to visualize ENP accumulation in *D. rerio* embryos and in *D. magna*. If combined with a visual image of the respective organism, the organ in which accumulation occurs can be determined. The increasing size of the ENPs leads to decreasing sensitivity of detection by LA-ICP-MS from soft biological tissue. As shown for sections of *D. rerio*, quantitative imaging by LA-ICP-MS is possible, when an external matrix-matched calibration is performed with the ENPs of similar size embedded in agarose gels. In this way, the extent of uptake of ENPs into organisms can be determined, internal exposure concentrations can be measured, and toxicological points of action can be identified. If the size distribution of ENPs in an organism differs from that of the initial ENP taken up by the organism, quantitative imaging becomes erroneous. It is then necessary to determine the size of ENPs in the organism for an adequate calibration and a subsequent quantification of the internal concentration of the ENP.

## Electronic supplementary material

ESM 1 (PDF 2542 kb)
