# Supplementary material for: Exploring LA-ICP-MS as a quantitative imaging technique to study nanoparticle uptake in Daphnia magna and zebrafish (Danio rerio) embryos
Source: Anal Bioanal Chem. 2015 May 6;407(18):5477–85. doi: 10.1007/s00216-015-8720-4 (PMC4477941; doi:10.1007/s00216-015-8720-4)
Supplement: Supplementary file 1 — (PDF 2542 kb) [file 216_2015_8720_MOESM1_ESM.pdf]

Analytical and Bioanalytical Chemistry

Electronic Supplementary Material

Exploring LA-ICP-MS as a quantitative imaging technique to study nanoparticle uptake in *Daphnia magna* and zebrafish (*Danio rerio*) embryos

Steffi Böhme, Hans-Joachim Stärk, Dana Kühnel, Thorsten Reemtsma

Materials and methods

Characterization of nanoparticle suspensions

The investigated silver and gold nanoparticle suspensions were provided and characterised in detail by partners of the EU-project NanoValid (**Tab. S1**). The three aluminum oxide nanomaterials (Al_2O_3-NPs) were purchased as powders from different industrial partners. These particles are named in the following as Alu1 (AEROXIDE ®, Alu C, Evonik Degussa GmbH), Alu2 (TAIMICRON ®, TM-DAR, Taimei Chemicals Co., LTD.) and Alu3 (NABALOX ®, NO-625-10, Nabaltec AG) and were already characterized in detail by the study of Böhme et al.[1].

Table S1 Chemical and physical properties of investigated nanomaterials

	**Characterization of powders**				**Characterization of suspensions**			
**Nano-material**	Coating	BET (m²/g)	X_{BET} (nm)	ρ (g/cm³)	X_{TEM} (nm)	X_{DLS} (nm)	Zeta-potential (mV)	Stabilizing agent
**AgNP**	PVP	-	-	10.49	21 ± 8	117 ± 24	-19	-
**AuNP**	Sodium citrate	-	-	19.3	13 ± 1	18 ± 2	-38	-
**Alu1** [1]	-	117	14	3.6	-	127	-58	SHMP
**Alu2** [1]	-	13.5	111	3.99	-	186	-64	SHMP
**Alu3** [1]	-	2	752	3.99	-	2500	-83	SHMP

ENP visualization and quantification by neb-ICP-MS and LA-ICP-MS

A quadrupole ICP-MS (ELAN DRCe, Perkin Elmer Sclex.) was applied to analyze the element concentrations of the exposed organisms, either from solution after acid digestion or from the aerosol generated by laser ablation. The exposed organism sections were ablated by a Nd:YAG laser (LSX 500, CETAC, USA). The laser ablation parameters can be found in the **Tab. S2**. The laser energy was adjusted to 60 % to ensure a complete ablation of the organic layer and a spot diameter of 50 µm was applied. The nebulizer was used in parallel to mix the laser aerosol with a blank solution and to have constant wet plasma conditions.

Table S2 Laser and ICP-MS parameters for the quantification and visualization of organism sections

Neb-ICP-MS		LA-ICP-MS	
ICP-MS parameters		ICP-MS parameters	
RF power (kW)	1.2	RF power (kW)	1.1
Lens voltage (V)	9.0	Lens voltage (V)	6.0
Argon nebulizer flow rate (L min^{-1})	0.9	Argon nebulizer flow rate (L min^{-1})	0.5
Argon auxiliary flow rate (L min^{-1})	1.2	Argon auxiliary flow rate (L min^{-1})	1.2
Argon makeup gas flow rate (L min^{-1})	0	Argon makeup gas flow rate (L min^{-1})	0.4
Dwell time (ms)	200	Dwell time (ms)	200
Measured isotopes (m/z)	^{45}Sc, ^{35}Cl, ^{66}Zn, ^{103}Rh, ^{27}Al, ^{107}Ag, ^{197}Au	Measured isotopes (m/z)	^{13}C, ^{27}Al, ^{107}Ag, ^{197}Au
		Laser parameters	
		Laser energy (%)	60
		Repitition rate (Hz)	20
		Laser spot size (µm)	50
		Distance between spots (µm)	50
		Delay between spots (sec)	35
		Scanning mode	spot raster
		Analysis time	3-4 h

Results and discussion

Visualization of ENP distribution by LA-ICP-MS

Within this study, a LA-ICP-MS method to visualize the nanoparticle uptake and the distribution in the biological tissues of environmental organisms, like the zebrafish (*Danio rerio*) embryo and *Daphnia magna* was developed. The 2D-color plots for Alu2 and Alu3 are shown in **Fig. S1**.

For ZFE most of the particles were accumulated at the chorion. On the contrary, the ENP distribution in *Daphnia magna* clearly indicates active uptake, since metal signals are elevated in the gut of the organisms and minor amounts are visualized to be accumulated in the gill and eye tissues.

The limit of detection (LOD) depends on the type of the introduced particle and the spot diameter which is used for the laser ablation itself (**Tab. S3**). In this context, an increasing spot diameter leads to an increase in the ablated area, a larger produced and evaporated volume and thus a higher detection limit. The LODs for silver and gold are lower when comparing them with the ones for aluminum, what is a result of the natural abundance of these elements which is higher for aluminum.

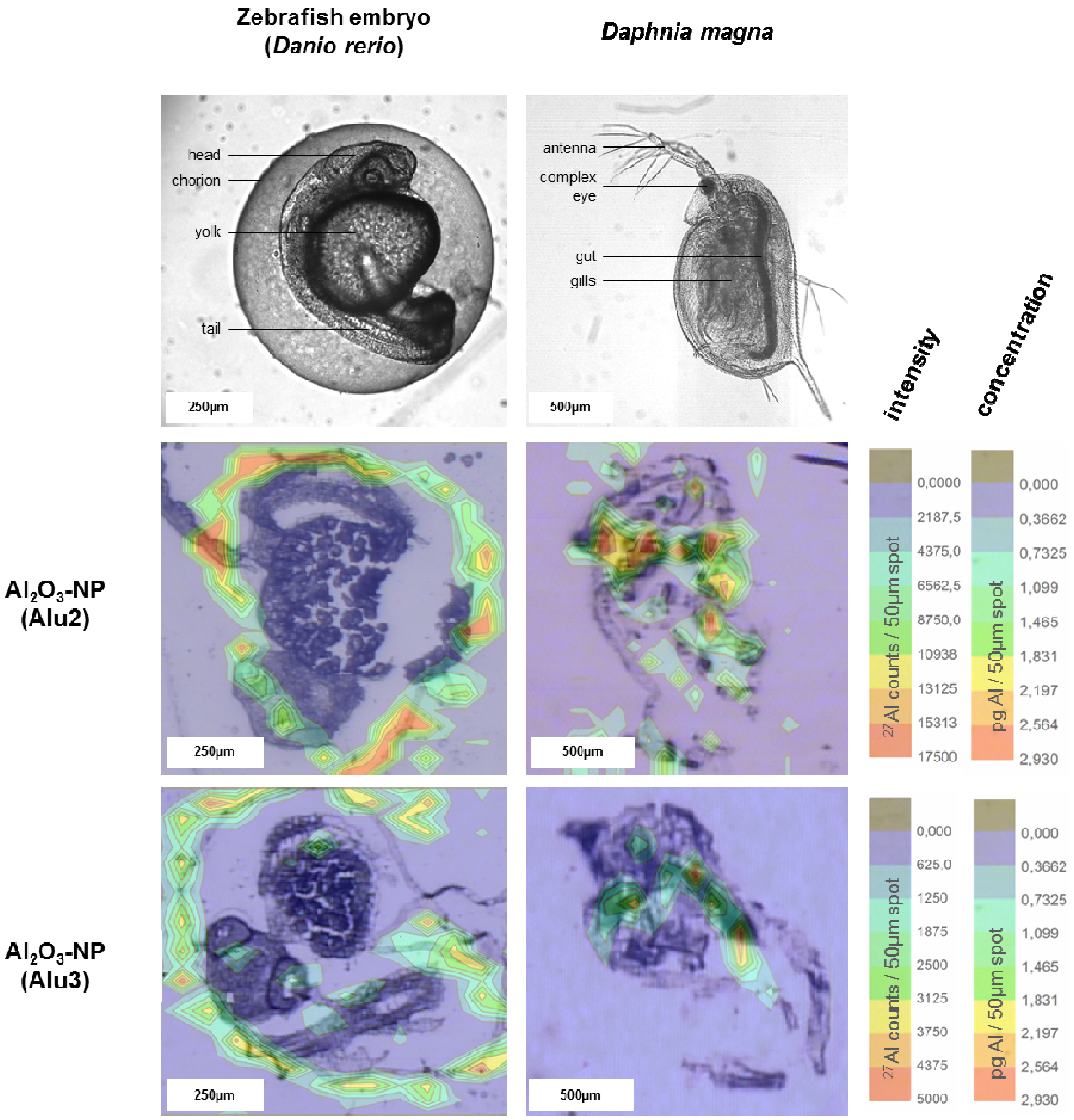

Fig. S1 Overlay of visual images and the signal intensity for the respective element as recorded by LA-ICP-MS of ZFE (left) and *Daphnia magna* (right)

Table S3 Limits of detection (LOD=Blank+3x standard deviation of the blank) for the different types of nanomaterials measured for the prepared matrix-matched standards

spot diameter	Al_2O_3 (mean for all types of particles)	AgNP	AuNP
50 µm	500-1000 fg Al / 50µm spot	35.7 fg Ag / 50µm spot	12.5 fg Au / 50µm spot

Matrix-matched calibration approach

To study a particle size-dependent effect in more detail, aerosol particles generated by LA-ICP-MS from Al_2O_3-NPs of different size from the agarose gels were collected approximately half the way between the ablation chamber and the plasma and investigated by SEM. The particle size distributions of the collected aerosols measured by SEM were in a comparable range as the initial sizes of the ENPs in suspension as determined by the BET method (x_{BET}) what is shown in **Tab. S4**. Furthermore, particle agglomerates or aggregates in the aerosol were observed which even exceeded the size of the original particles.

Table S4 Comparison of initial particle sizes and particle sizes determined with SEM after laser ablation

Nanomaterial	Initial primary particle size of the particle suspension (nm) [1]	Particle size detected after laser ablation (nm)	Size of agglomerates after laser ablation (nm)
Alu1	14	35 ± 11	239 ± 9
Alu2	111	48 ± 29	375 ± 130
Alu3	752	503 ± 367	3420 ± 1360

Quantification of ENP in ZFE and *Daphnia magna* by matrix-matched calibration

For the comparison and validation of LA-ICP-MS results, the uptake concentrations were additionally determined by acid digestion and subsequent neb-ICP-MS analysis of whole organisms. The blank values of both methods are listed in **Tab. S5** whereby no differences between the two organism species were observed.

Table S5 Blank values of untreated organisms determined by neb-ICP-MS and LA-ICP-MS (n≥3)

Nanomaterial	µg element / L	ng element / organism
	neb-ICP-MS	LA-ICP-MS
AgNP	0.31 ± 0.09	0.32 ± 0.32
AuNP	0.36 ± 0.04	0.26 ± 0.06
Al_2O_3 (Alu1)	1.01 ± 0.10	0.78 ± 0.11
Al_2O_3 (Alu2)	1.00 ± 0.02	0.89 ± 0.12
Al_2O_3 (Alu3)	1.37 ± 0.53	2.36 ± 0.30

References

(1) Böhme, S.; Stärk, H.-J.; Meißner, T.; Springer, A.; Reemtsma, T.; Kühnel, D.; Busch, W. *J Nanopart Res* **2014**, *16*, 1-15.
